# Predicting the spatial organization of chromosomes using epigenetic data

**DOI:** 10.1186/s13059-015-0752-8

**Published:** 2015-08-29

**Authors:** Raphaël Mourad, Olivier Cuvier

**Affiliations:** Laboratory of Molecular Biology of Eukaryotes (LBME), CNRS - University of Toulouse (UPS), F-31000 Toulouse, France

## Abstract

Chromosome folding can reinforce the demarcation between euchromatin and heterochromatin. Two new studies show how epigenetic data, including DNA methylation, can accurately predict chromosome folding in three dimensions. Such computational approaches reinforce the idea of a linkage between epigenetically marked chromatin domains and their segregation into distinct compartments at the megabase scale or topological domains at a higher resolution.

Please see related articles: http://dx.doi.org/10.1186/s13059-015-0741-y and http://dx.doi.org/10.1186/s13059-015-0740-z

## Introduction

The ability to probe the spatial organization of chromosomes through the combination of chromosome conformation capture methods with high-throughput sequencing (3C–Hi-C) has revealed how chromosomes organize into active and inactive compartments (indicated ‘A’ and ‘B’ , respectively) [1]. This pioneer work highlighted how the fractal organization of chromosomes could favor their folding into individual domains and revealed topologically associating domains (TADs) and sub-TADs (reviewed by Tanay and Cavalli [2]), which represent a pervasive structural feature of the organization of the genome. TADs favor specific long-range contacts between regulatory elements pertaining to the same domain. Such three-dimensional organization of chromosomes into spatially distinct domains sheds light on how complex genomes might set specific transcriptional programs to regulate genes individually or as groups of genes.

Specific long-range contacts formed between enhancers or repressors and their cognate promoters are confined within TADs through additional elements, named insulators or chromatin boundaries, that border TADs [3–6]. Insulator proteins, including CCCTC-binding factor (CTCF), bind and then recruit the architectural factor cohesin that stabilizes DNA loops formed between distant elements inside TADs, thereby preventing enhancers from targeting ectopic promoters outside of such domains [3–5].

The three-dimensional organization of chromosomes into TADs corresponds with epigenetically defined domains that are marked by specific histone modifications [2]. Such three-dimensional folding of chromosomes participates in the ‘setting’ of epigenomes, specifically in human cell lineages [7]. Integration of the massive amounts of epigenomic data, including DNA methylation and histone modifications, thus represents a major hurdle for understanding how chromatin organization governs cell identity epigenetically.

## Modeling chromosomes in three dimensions

Although TADs represent a pervasive structural feature of genome organization, approximately one-third of them define more-labile structures that change significantly upon cellular differentiation [7]. Understanding how multiple hierarchical levels of genome organization impact on epigenetic (re-)programming might thus largely rely on integrating Hi-C data together with epigenomic data through the development of bona fide computational approaches [2, 8]. The machine learning technique known as ‘random forests’ applied to genomic data profiling histone modifications by means of chromatin immunoprecipitation and sequencing (ChIP-Seq) has recently allowed the prediction of Hi-C matrices and TAD borders [7]. Such approaches might thus help to unravel the nature and dynamics of epigenomes during cellular differentiation at the molecular level.

### Epigenome-based prediction of A and B compartments in the nucleus

Dekker and collaborators originally showed that chromosomes are spatially segregated into three-dimensional compartments A and B, respectively [1, 8]. These compartments are cell type-specific, and they strongly associate with euchromatin and heterochromatin, respectively. Two papers take this further. In this issue of *Genome Biology,* Fortin and Hansen report how they have used such a principle to predict chromosomal compartments from epigenetic domains as defined by DNA methylation data [9]. High methylation in a gene promoter is known to silence the expression of the corresponding gene. Fortin and Hansen successfully predicted A and B compartments in different human cells. For this purpose, they first normalized the Hi-C contact matrix to remove the effect of polymer distance, calculated the correlation matrix and used principal component analysis [9]. The resulting first principal component distinguishes compartments A and B. Then the authors sought to predict this principal component from methylation data assayed using the available Illumina 450 k microarray platform. Notably, the authors computed a correlation matrix from Illumina 450 k data that highlighted long-range correlations among methylation profiles obtained from different samples. In addition, their methylation-based correlation matrix provided a good prediction accuracy for the Hi-C correlation matrix (*R* = 0.85) compared with using the average methylation data profile (*R* = 0.56). A higher accuracy was also obtained (*R* = 0.93) after excluding hard-to-classify genomic loci at the borders of A and B compartments. The authors successfully achieved high prediction accuracy using other epigenetic data such as those resulting from DNase hypersensitivity. Finally, the authors showed the functional implications of such compartment predictions by emphasizing the link with the somatic mutation rate, which is lower in compartment A. Such work is in line with recent data that demonstrated a key role for the three-dimensional organization of chromosomes in setting epigenome landscapes in human cell lineages [7].

### Predicting TADs and chromatin interaction hubs

In a second associated paper in this issue, Huang and colleagues proposed a similar approach to predict TADs by using epigenetic data from various human cell lines, including tumor cells [10]. They used a state-of-the-art computational classifier — Bayesian additive regression trees (BART) — that successfully predicted the presence of TAD borders from the localization of histone modifications or of CTCF insulator protein as inferred from ChIP-Seq data, with a good prediction accuracy (area under the curve (AUC) = 0.77). CTCF emerged as the best predictor in the model, in agreement with recent data demonstrating its role in TAD formation [4, 5]. In their work, Huang and collaborators also predicted the location of chromatin hubs that play an important role in gene regulation. Chromatin hubs seem to represent complex genomic loci where multiple long-range interactions cluster a number of distant regulatory elements together with the nearby genes. Of note, the authors could show the BART classifier predicted the location of such hubs with high prediction accuracy (AUC = 0.87) [10], which will be of potential significance for unraveling complex genetic disorders.

## General implications

More than 20,000 DNA methylation samples are readily available at Gene Expression Omnibus (GEO) and The Cancer Genome Atlas (TCGA), which might serve to predict three-dimensional chromosome contact maps through approaches similar to those developed by Fortin and Hansen [9]. Computational methods integrating epigenomes and Hi-C data clearly represent formidable tools to guide further in-depth analysis of the role of chromosome organization in cell identity [2, 7, 8]. Disease-associated and trait-associated epigenetic variants generated by ENCODE and NIH Roadmap Epigenomics consortia and haplotype-resolved epigenome data have further revealed allele-specific regulatory mechanisms through long-range contact maps during lineage specification [7], which paves the way for understanding the molecular basis of human disease.

Computational approaches contribute to a promising avenue of research in human genetics aiming to unravel key aspects of epigenome regulation through chromosome folding. Fortin and Hansen found long-range correlations among methylation profiles of distant loci, highlighting a coordinated regulation of DNA methylation through three-dimensional clustering of methylated islands. A remaining question is the identity of the molecular drivers of such functional long-range contacts. Our understanding of the regulatory mechanisms of cellular identity, differentiation or reprogramming could thus depend largely on how long-range contacts in chromatin are regulated [7]. Such regulatory events probably involve an interplay between epigenetic regulators and CTCF, cohesin or additional architectural proteins [3, 4, 6, 7].

## Concluding remarks

The papers by Fortin and Hansen and by Huang and colleagues represent successful attempts to predict from epigenetic data higher-order chromatin folding features such as compartments and TADs [9, 10]. Further development of computational approaches using more-sophisticated models such as those derived from polymer physics or machine learning should help to improve prediction of Hi-C matrices [2, 8]. Another major goal is to reconstruct two-dimensional contact maps aiming at unraveling the molecular basis of long-range contacts through aggregation of Hi-C data [6]. Future models should also integrate epigenomic data together with knowledge of the cognate ‘writer’ , ‘reader’ and ‘eraser’ epigenetic factors over the cell cycle. Finally, understanding epigenome propagation might require monitoring the turnover rates of epigenetic marks, which is what conditions ‘epigenetic memory’, along with the dynamics of long-range contacts.

## Abbreviations

3C–Hi-C: Chromosome conformation capture and high-throughput sequencing
AUC: Area under curve
BART: Bayesian additive regression trees
ChIP-Seq: Chromatin immunoprecipitation and high-throughput sequencing
CTCF: CCCTC-binding factor
TAD: Topological associating domains
